# Modified micromarsupialization technique as an alternative primary treatment for ranulas: A case series in a resource‐challenged economy

**DOI:** 10.1002/cre2.627

**Published:** 2022-10-05

**Authors:** Mutassim Elnager, Samuel E. Udeabor, Abosofyan S. A. Elfadeel, Chidozie I. Onwuka, Mashail M. M. Hamid, Yassin M. A. Alsubaie

**Affiliations:** ^1^ Department of Oral and Maxillofacial Surgery, College of Dentistry King Khalid University Abha Saudi Arabia; ^2^ Department of Oral and Maxillofacial Surgery Khartoum Teaching Dental Hospital Khartoum Sudan

**Keywords:** case series, modified micromarsupialization, sublingual ranula

## Abstract

**Objectives:**

Ranula is one of the commonest salivary gland cysts that mostly occur due to mucus extravasation from the sublingual salivary gland. Treatment of this lesion is still somewhat shrouded in controversy and varies from conservative treatment to surgical excision of the causative gland.

**Materials and methods:**

This was a case series in quasi‐experimental design that evaluated the outcome and complications of the modified micromarsupialization technique as a newly introduced treatment at our center for simple sublingual ranula over a 2‐year period.

**Results:**

Twenty patients were treated by the modified micromarsupialization technique and followed up for 1 year. There was complete resolution following this technique in 17 patients (85%) with no evidence of recurrence or complications, whereas 2 patients (10%) showed partial resolution and the remaining 1 patient (5%) showed a failure and recurrence. The age of the patient, the size of the ranula, and the retention of sutures throughout the study period did not significantly affect the treatment outcome.

**Conclusions:**

The modified micromarsupialization technique was a simple and effective treatment that should be used as a primary treatment option for simple ranulas and we recommend it to be the first‐choice treatment before surgical excision of the sublingual gland, especially in a resource‐challenged economy like ours.

## 1 INTRODUCTION

Ranulas are bluish cystic enlargements in the floor mouth, which are usually painless, dome‐shaped, and resemble the underbelly of a frog (Sandrini et al., 2007; Yuca et al., 2005). They are either mucus retention cysts in the sublingual gland ductal system or, more commonly, mucus extravasation cysts formed as a result of ductal disruption (Macdonald et al., 2003). Ranulas are classified into sublingual, plunging, and sublingual plunging depending on their location (Horiguchi et al., 1995). Sublingual ranulas are formed on the floor of the mouth, whereas the plunging ranulas develop as soft, cystic swellings in the submandibular or upper cervical regions (Horiguchi et al., 1995). In some instances, the sublingual ranulas extend through the mylohyoid muscle to the cervical region and are thus termed sublingual plunging ranulas (Horiguchi et al., 1995).

The etiology of ranulas has remained largely unknown, but they have been described in relation to trauma (including surgical traumas; Dietrich et al., 2011), congenital anomalies (Morton et al., 2010), and diseases of salivary glands, especially the sublingual gland (Takagi et al., 2021).

Different treatment modalities have been adopted for the management of ranulas. Conservative and nonsurgical management that avoids the risks of surgical complications and morbidities has been favored by some clinicians (Brannan et al., 2019; Nguyen & Orloff, 2017; Ryu et al., 2017). Sclerotherapy is one of the nonsurgical means of ranula treatment that is being advocated with the use of different sclerosing agents like OK‐432 (Picibanil; Roh & Kim, 2008) and ethanol (Nguyen & Orloff, 2017; Ryu et al., 2017). Intra‐cystic injections of steroids (Bowers & Schaitkin, 2021) and liquid nitrogen (cryotherapy; Garg et al., 2014) have also been reported by some surgeons. All these nonsurgical treatments of ranulas have yielded varying degrees of success.

Despite the successes touted by these nonsurgical approaches, some of the chemicals and solutions used for the nonsurgical approaches are not readily available and, where available, cannot be afforded by the majority of patients in third‐world countries. Simple surgical excision of the sublingual gland and the excision of the associated ranula has therefore remained the commonest traditional means of ranula treatment (Garg et al., 2014; Lee et al., 2015). Surgical excision, however, is riddled with morbidities and complications that range from recurrences to damage to vital structures such as the lingual nerve and the submandibular duct (Moraes et al., 2015). Surgeons therefore are adopting various minimally invasive surgical approaches to the management of this lesion, in order to limit or minimize the aforementioned complications.

One of these minimally invasive surgical techniques is the modified micromarsupialization technique. This technique basically utilizes sutures carefully placed in different sites of the ranula and retained for a period to encourage the formation of many epithelialized tracts that further promote mucous drainage and eventual resolution of the ranula (Matondkar et al., 2019; Sandrini et al., 2007).

This study was therefore designed to assess the treatment outcome of the use of a modified micromarsupialization technique for patients with simple sublingual ranulas (SSRs) that presented to our hospital.

## 2 PATIENTS AND METHODS

This study was carried out in the Oral and Maxillofacial Surgery clinic of Khartoum Teaching Dental Hospital, Khartoum, Sudan, after obtaining the institution's ethical clearance. Consecutive patients attending our outpatient clinic with clinically diagnosed SSR were included in this study. It was a case series study with no randomization or control group. All the patients who presented within the study period of 2 years, met the inclusion criteria, and consented to the study were included.

### 2.1 Patients selection

Patients who were clinically diagnosed with SSR and had not received any form of ranula treatment were selected. They were then given the option of the routine standard treatment of ranula in our center, which is surgical excision of the involved sublingual gland or the new treatment option of modified micromarsupialization technique. Patients who chose the new treatment modality and agreed to participate in the study were then selected and included in the study.

### 2.2 Procedure

The entire procedure was done as a minor oral surgical procedure on an outpatient basis under local anesthesia.

**Figure 1:**
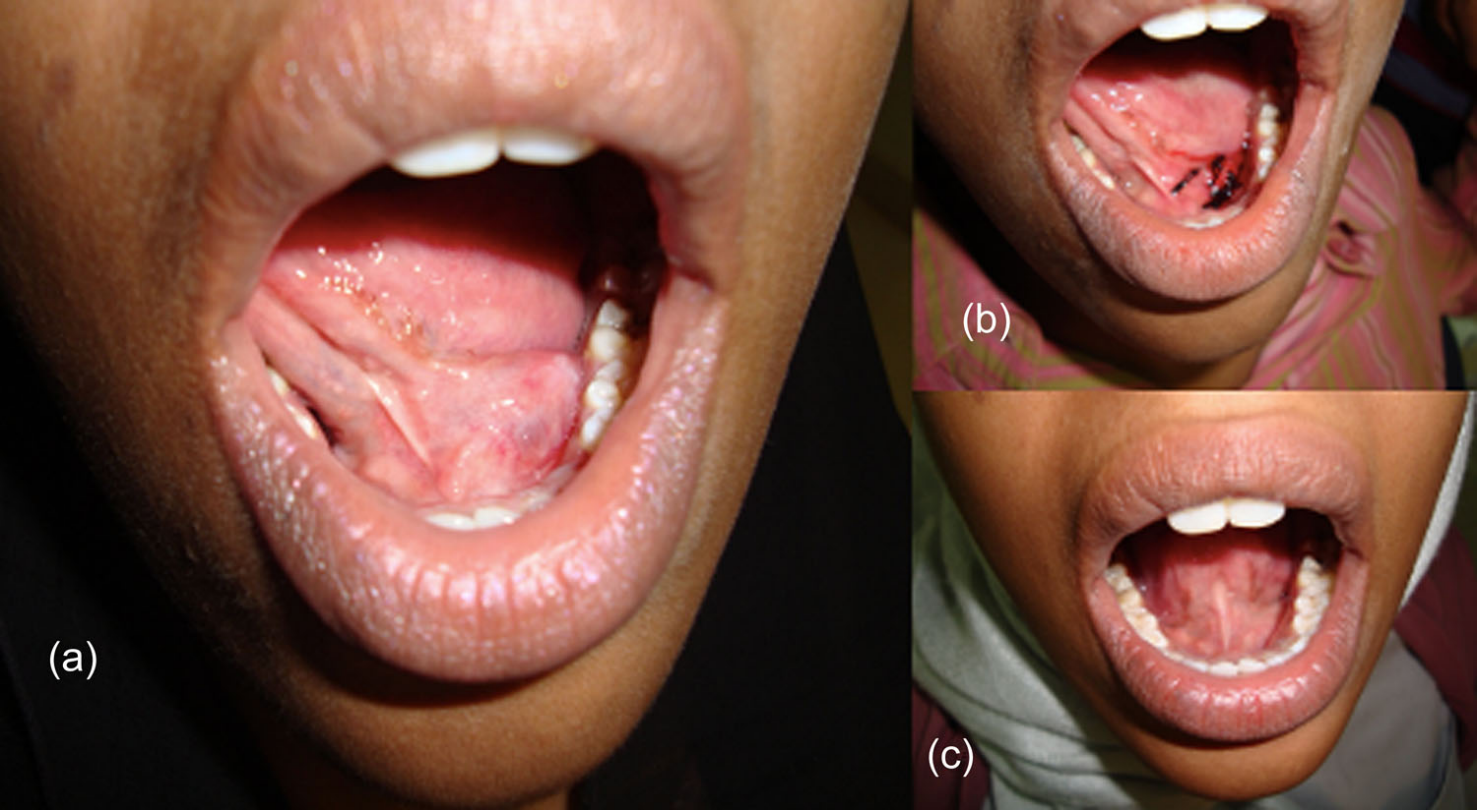
Ranula patient: (a) Preoperative. (b) Postoperative with sutures. (c) 1‐year follow‐up showing complete resolution.

The mouth was first rinsed with 2% Chlorhexidine solution and a lingual nerve block of the affected side was administered. Additional conscious sedation was used for uncooperative child patients. Interrupted sutures (3.0 black silk suture) were then passed superficially through the dome of the ranula. Noncutting needle was used to avoid tissue tears. Additionally, the suture was manipulated in and out of the lesion after passing the needle to ensure the establishment of a proper drainage channel before the knot was tied. The number of interrupted sutures passed depended on the size of the ranula and, in some cases, up to six interrupted sutures were placed (Figure 1). The suture knot was not too tight to avoid necrosis. Further, care was taken not to ligate the Warton's duct, as this could lead to acute sialoadenitis of the submandibular gland. All the patients treated tolerated the procedure with no adverse effects throughout the follow‐up period. Patients were given oral hygiene instructions and followed up weekly for the next 30 days to review treatment progress and monitor compliance with proper oral hygiene.

The sutures were left in place until a spontaneous loss and any suture that remained was removed on the 30th day of the visit. The patients were subsequently followed up at 3 monthly intervals for 1 year. All the patients were treated by the main researcher, to ensure the internal validity of the procedure.

The data collected were analyzed by descriptive statistics using SPSS version 25 and the results were presented in graphs, tables, and charts.

## 3 RESULTS

During the 2‐year study period, 20 patients diagnosed with SSR were treated by the modified micromarsupialization technique in our center. Nine of these patients (45%) were male, whereas 11 (55%) were females and their ages ranged from 4 years to 46 years. The 6–15 years age bracket was the most affected (45%). Four (20%) reported a history of trauma leading up to the ranula formation, 10 (50%) were located on the right floor of the mouth, whereas 8 (40%) were found on the left and 2 (10%) were bilateral. The sizes range from <1 cm^3^ in seven patients (35%), 1–2 cm^3^ in six patients (30%), 2–3 cm^3^ in one patient (5%), to >3 cm^3^ in six patients (30%; Table 1).

**Table 1:** Clinical presentation of ranula patients

	Frequency	Percent (%)
History of trauma		
Yes	4	20%
No	16	80%
Site of ranula		
Right	10	50%
Left	8	40%
Bilateral	2	10%
Size of ranula		
1 cm^3^ or below	7	35%
1–2 cm^3^	6	30%
2–3 cm^3^	1	5%
>3 cm^3^	6	30%
Total	20	100%

**Figure 2:**
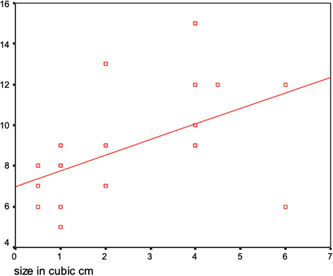
Linear regression graph showing the relationship between the size of the ranula and the number of sutures.

The number of sutures placed was directly proportional to the size of the ranula and throughout this trial, the maximum number of sutures were applied to obtain better results. From our patients' calculations, linear regression showed that a relationship could be obtained by using the following equation (*y* = 6.9*x*+ 0.5), wherein *y* is the size of the ranula in cubic cm and *x* represents the number of sutures (Figure 2).

During the first 30‐day follow‐up, only 7 (35%) patients retained the full complement of the original sutures placed up till their removal on the 30th day.

**Figure 3:**
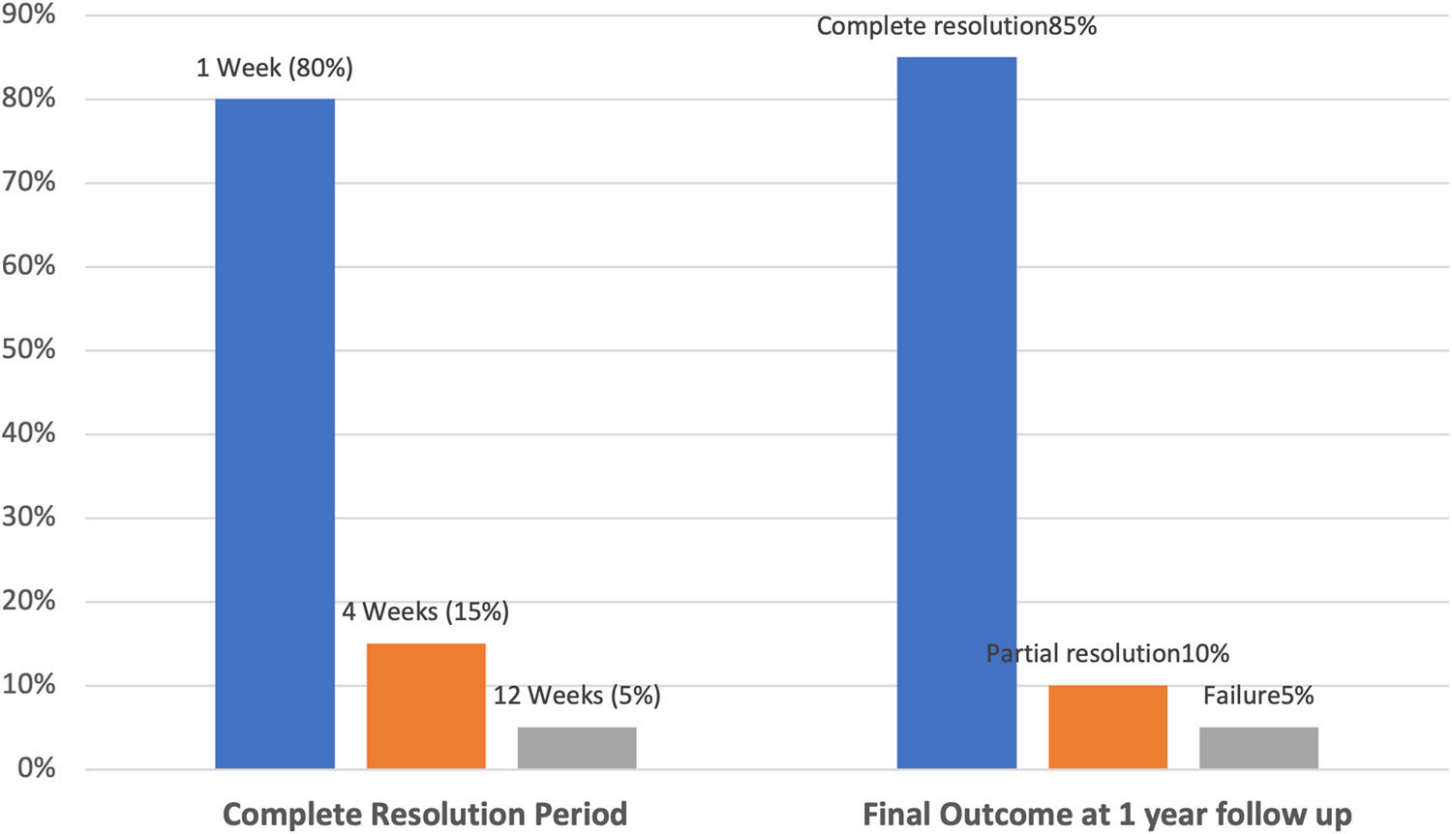
Complete resolution period and final outcome at 1‐year follow‐up.

Most of the patients (80%) achieved complete resolution of the ranula after 1 week, 3 patients (15%) at 4 weeks, and only 1 patient (5%) showed delayed complete resolution up to 12 weeks of follow‐up (Figure 3).

After 1 year of follow‐up, there was complete resolution in 17 patients (85%) with no evidence of recurrence or complications, whereas 2 patients (10%) showed partial resolution and the remaining 1 patient (5%) showed a failure and recurrence (Figure 3).

There was no significant correlation between age and outcome (*p* = .679; Table 2). Also, the outcome bears no significant correlation to the size of the ranula before treatment (*p* value = .749) (Table 3).

**Table 2:** Association between age and the final outcome

	Complete resolution	Partial resolution	Failure	Total
5 Years and below	3 (75%)	1 (25%)	–	4 (100%)
6–15 Years	8 (88.89%)	–	1 (11.11%)	9 (100%)
16–30 Years	4 (80%)	1 (20%)	–	5 (100%)
Above 31 Years	2 (100%)	–	–	2 (100%)
Total	17 (85%)	2 (10%)	1 (5%)	20 (100%)

* *χ* ^2^ test performed, *p* = .697 (not significant).

**Table 3:** Association between the size of ranula and final outcome

	Complete resolution	Partial resolution	Failure	Total
1 cm^3^ or below	6 (85.71%)	1 (14.29%)	–	7 (100%)
1–2 cm^3^	5 (83.33%)	–	1 (16.67%)	6 (100%)
2–3 cm^3^	1 (100%)	–	–	1 (100%)
More than 3 cm^3^	5 (83.33%)	1 (16.67%)	–	6 (100%)
Total	17 (85%)	2 (10%)	1 (5%)	20 (100%)

* *χ* ^2^ test performed, *p* = .749 (not significant).

The complete resolution period and the effect of early loss of sutures were correlated and showed that in patients with no early suture loss, 85% had a complete resolution in 1 week and 15% in 1 month, whereas patients with early suture loss showed 76% complete resolution in 1 week, 15% in 1 month, and 9% in 3 months, respectively. The *χ* ^2^ test of this correlation yielded no statistical significance with a *p* of 0.746. (Table 4).

**Table 4:** Association between early loss of sutures and complete resolution period

	Week	1 Month	3 Months	Total
No stitches were lost during the first week	6 (85.71%)	1 (14.29%)	–	7 (100%)
Stitches lost during the first week	10 (76.92%)	2 (15.38%)	1 (7.69%)	13 (100%)
Total	16 (80%)	3 (15%)	1 (5%)	20 (100%)

* *χ* ^2^ test performed, *p* = .746 (not significant).

## 4 DISCUSSION

The modified micromarsupialization technique when used for selected cases of ranulas, as in this study, cases of SSRs, promises to be an effective and simpler alternative to the traditional treatment method of sublingual gland excision. It is a minimally invasive surgical procedure that avoids most of the surgical morbidities and complications associated with the standard surgical treatment. In this regard, it is similar to the nonsurgical means of ranula management like sclerotherapy (Roh & Kim, 2008) and cryotherapy (Garg et al., 2014), which utilizes different chemicals and solutions to achieve the resolution of the ranula while at the same time delivering little or no complications. However, some of these chemicals and solutions are difficult to access in third‐world countries and struggling economies such as ours, and, where available, are too expensive and out of reach to our regular patients. It, therefore, makes the modified micromarsupialization technique a more effective and cheaper alternative for these patients.

One of the major limitations in the use of this technique, however, is that it is case sensitive and can only be used for a few selected cases of ranulas. Patients with plunging ranulas for example still have to undergo surgical excision of the lesion and the involved salivary gland.

Our study cohort ranged in age from 4 to 46 years with most of them (45%) in the 6–15 years age bracket. This agrees with previous studies that reported ranulas to mostly develop in the first two decades of life (Brannan et al., 2019; Matondkar et al., 2019; Roh & Kim, 2008). The results of this present study, however, showed that the outcome of this surgical technique was not influenced by the age of the patient at presentation. It can therefore be used in children, adolescents, and adults.

Some authors believed that this technique should not be applied to large ranulas that fill the floor of the mouth, as this will not guarantee a successful outcome (Goodson et al., 2015). However, our study reached a different conclusion on this subject. We used this technique for all the sublingual ranulas that presented to us despite the size, as long as they are limited to the floor of the mouth without plunging through the mylohyoid muscle. Our findings showed that size did not affect the outcome, therefore suggesting that this technique could be used for both large and small sublingual ranulas occupying the floor of the mouth.

We made slight modifications to the original modified micromarsupialization technique as proposed by Sandrini et al. (2007). We used lingual nerve block as opposed to topical anesthesia as this was better tolerated by our patients, especially the children. Additionally, we used 3.0 instead of 4.0 black silk suture: we are of the opinion that larger‐sized suture material would lead to better‐epithelialized tracts that would encourage good and continuous mucus drainage to prevent recurrences.

Additionally, we used the maximum number of sutures possible to encourage the development of multiple fistulated tracts that would lead to a higher success rate. The number of sutures used by previous surgeons was usually arbitral. We therefore developed the following formula based on the findings from our patients: *y* = 6.9*x* + 0.5 in which *y* is the size of the ranula in cubic cm and *x* represents the number of sutures. This is intended to serve as a guide on the approximate number of sutures to be applied on a case‐by‐case basis to achieve the best outcome.

Finally, we manipulated the suture in and out of the lesion after passing the needle to ensure the establishment of a proper drainage channel before tying the knot.

## 5 CONCLUSION

The modified micromarsupialization technique in the management of SSR in our center yielded a very good outcome in our study sample. This technique proved to be a simple, safe, less time‐consuming, cheap, and effective procedure to treat SSR patients on an outpatient basis.

Our study also found out that the age of the patient and the size of the ranula at presentation did not affect the final outcome when treated by this technique. We would therefore recommend this technique as a primary and first‐line management technique for SSRs in both children and adults, especially in resource‐challenged environments and economies like ours.

## AUTHOR CONTRIBUTIONS

Mutassim Elnager and Yassin M. A. Alsubaie conceived the study. Mutassim Elnager, Samuel E. Udeabor, Abosofyan S. A. Elfadeel, Chidozie I. Onwuka, and Mashail M. M. Hamid designed the methodology. Mutassim Elnager performed all the surgical procedures. Samuel E. Udeabor and Mutassim Elnager prepared the original draft. Mutassim Elnager, Samuel E. Udeabor, Abosofyan S. A. Elfadeel, Chidozie I. Onwuka, Mashail M. M. Hamid, and Yassin M. A. Alsubaie reviewed and edited the entire manuscript.

## CONFLICTS OF INTEREST

The authors declare no conflicts of interest.

## ETHICS STATEMENT

The study was conducted according to the guidelines of the Declaration of Helsinki and approved by the Institutional Review Board of Khartoum Teaching Dental Hospital, Khartoum Sudan (IRB/KTDH/2018/0010).

## Data Availability

The data that support the findings of this study are available from the corresponding author upon reasonable request.
